# Baden-Baden

**Published:** 1890-04-19

**Authors:** 


### Baden-Baden.

The recent departure of her Majesty for Aix-les-Bains, and the reports in circulation earlier in the year as to her intended visit to Homburg, which proved unfounded, have drawn attention to German and French spas. After Homburg Baden-Baden is the principal spa of Germany. During the spring it may be likened to a dismantled fortress, for the season only begins in May. But already there is a fair share of visitors hailing, as the lists inform us, from Hamburg on the one hand to Hungary on the other. Anglo-Americans are mostly on the Riviera. The twenty or thirty medical men, whose names with addresses and hours of consultation appear in the papers, cannot, however, as yet, hope for a very numerous clientèle. Quite lately there was snow on the hills around the town, and the weather was bleak and raw. The theatre was closed all the week but Wednesday. The concerts in the Conversations-house were poorly attended, and the modest visitors seated themselves behind as if to leave room in front for the great who were expected, but did not come. These entertainments are given daily, sometimes twice a day, admission being by tickets, costing a mark, that is a shilling, which admit to concert, grounds, and reading-room, open throughout the day.

Close by is the Trink-Halle, where the waters are taken. These flow from a fountain in the centre of the hall, supplied by the Hauptstollen spring. They are rich in chloride of sodium, and contain besides considerable quantities of chloride of potassium, sulphate and carbonate of calcium, and chloride of lithium. The temperature is 145 deg. Fahr. Then there are the Frederick baths, open from May 1st to September 30th, between the hours of 6 a.m. and 6 p.m., and during the rest of the year from 8 a.m. to 4 p.m. From tub-baths, costing about 8d. each, to electric baths, costing 3s., and only to be taken in the presence of a physician, all are provided in this institution. Inhalations of so-called pulverized mineral waters, and the vapours of mineral waters, may likewise be prescribed. Cases of uric acid diathesis, gout, scrofula, sequelæ after accident, certain kidney, nervous, and skin diseases, anæmics and convalescents are especially benefited by this course of treatment.

Some of the hotels are first-class, and very expensive; others of the second rank, and very cheap. In the latter a first-floor bedroom may be secured for 1s. 6d. per night, but there will be no carpet, and the cooking will be very trying to an English palate. Boarding-house prices for pension range from 5s. per diem, but breakfast consists of a roll and coffee, and after the evening meal visitors go supperless to bed. Furnished apartments may be hired in the numerous mansions, meals being taken in the restaurants of the town.

Excellent accommodation is provided in the Sanatorium just completed. A couple of unimportant details appear, however, worthy of notice. The heating apparatus of the elegantly-furnished rooms is arranged in a corner into a system of pipes standing upright, and decorated, so as to simulate a small organ. The scraper-mats outside the doors are formed of wire spirals set in frames, at once grateful to the feet, and very efficient. The following extracts may be made from the rules drawn up in English, French, and German. Breakfast is served from 8—9 a.m., dinner at 1 p.m., the evening meal at 7 p.m., the meals being taken all together in the common dining-room. No beer is served at dinner or in public rooms, with the exception of the billiard and smoking rooms. Wines are provided from the cellars; visitors bringing their own must pay one shilling per bottle corkage. The house physician visits each patient daily; those desiring a special visit must drop their cards into his box. The Sanatorium closes at 11 p.m. Each flat possesses a handsome balcony, with an ample view across the valley, in which nestles the little capital of the Black Forest.

